# Insulin-Related Biomarkers to Predict the Risk of Metabolic Syndrome

**DOI:** 10.5812/ijem.10418

**Published:** 2013-10-11

**Authors:** Tomoyuki Kawada

**Affiliations:** 1 Department of Hygiene and Public Health, Nippon Medical School, Tokyo, Japan

**Keywords:** Insulin Resistance, Biomarkers, Metabolic Syndrome X, Aging

## Abstract

**Background:**

The predictive ability of insulin resistance or insulin sensitivity, in combination with traditional cardiovascular risk factors for metabolic syndrome (MetS), has not yet been clearly evaluated in Japanese male subjects.

**Objectives:**

A one-year follow-up study was conducted to determine the ability of the insulin-related biomarkers to predict the risk of MetS development.

**Patients and Methods:**

A total of 2642 male workers of a Japanese company free from MetS at the baseline were monitored. The homeostasis model assessment for insulin resistance (HOMA-IR), and quantitative insulin sensitivity check index (QUICKI) were selected as the insulin-related markers.

**Results:**

The incidence of metabolic syndrome after one year was 8.8%. A multiple logistic regression analysis identified regular physical activity, age (≥ 45 years old), serum uric acid (≥ 7 mg/dL), serum alanine aminotransferase (≥ 45 IU/L), serum C-reactive protein (≥ 0.1 mg/L) and HOMA-IR (≥ 2.5) as significant risk factors for the development of MetS, with odds ratios (95% confidence intervals) of 0.68 (0.50 – 0.92), 2.0 (1.5 – 2.6), 2.2 (1.6 – 3.0), 1.5 (1.02 – 2.2), 1.4 (1.01 – 2.0), and 2.3 (1.6 – 3.3), respectively. When QUICKI was used instead of HOMA-IR, age (≥ 45 years old), serum uric acid (≥ 7 mg/dL), serum gamma-glutamyl transferase (≥ 50 IU/L), and QUICKI (≤ 0.33) were identified as significant contributors to the risk of MetS, with odds ratios (95% confidence intervals) of 0.68 (0.51 – 0.93), 2.0 (1.5 – 2.6), 2.2 (1.6 – 3.0), 1.4 (1.01 – 2.0), and 2.5 (1.7 – 3.6), respectively.

**Conclusions:**

The mathematical meaning of the two insulin-related biomarkers examined was the same, and the odds ratios of the two biomarkers were almost the same after adjustments for other independent variables.

## 1. Background

Insulin resistance is considered to be a risk factor for the development of metabolic syndrome (MetS). A recent review has elucidated the genetic background for the cause-effect relationship between these pathological conditions (1). In addition, the serum C-reactive protein (CRP), widely used as an indicator of systemic inflammation is associated with insulin resistance and the risk of MetS development (2).

Serum gamma-glutamyl transferase (GGT) has been used as an indicator of alcohol overload and several hepatic inflammatory diseases, and is considered as a useful biomarker of the risk of MetS or cardiovascular disease (3, 4). In addition, serum alanine aminotransferase (ALT) is also a predictive biomarker for MetS (5).

Hyperuricemia is also a risk factor for MetS, which has been explained based on the insulin resistance, visceral fat accumulation or xanthine oxidoreductase metabolism (6-8).

## 2. Objectives

The author conducted a one-year follow-up study to investigate the predictive ability of two insulin-related biomarkers, namely, the homeostasis model assessment for insulin resistance (HOMA-IR), and quantitative insulin sensitivity check index (QUICKI) (9-12), in combination with some other risk factors, as independent variables, for identifying the risk of MetS development.

## 3. Patients and Methods

**Figure 1.**
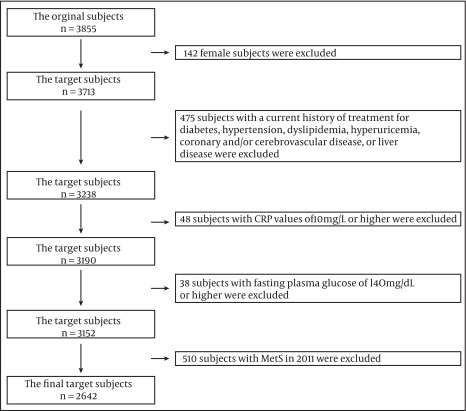
A Flow-Chart for the Study Population

A total of 3713 male workers (age range, 35 – 63 years) of a Japanese company were recruited for this study. All the subjects had responded to a questionnaire containing questions on the current medical and treatment history and some lifestyle factors. They underwent two consecutive annual health examinations, which included measurements of the waist circumference (WC) and systolic/diastolic blood pressure (measured in the sitting position after the subjects had rested for 3 minutes), and collection of fasting blood samples. Subjects with a current history of treatment for diabetes, hypertension, dyslipidemia, hyperuricemia, coronary and/or cerebrovascular disease, and liver disease were excluded (n = 475). Subjects with CRP values of 10 mg/L or higher were excluded by considering acute occult inflammation or chronic infectious disorder (n = 48), and subjects with MetS or high plasma glucose levels (>= 140 mg/dL) at the baseline study were also excluded to maintain the validity of the results on the insulin-related biomarkers (n = 38). Namely, as a simple surrogate index for insulin sensitivity/resistance, I adopted the upper limitation value of plasma fasting glucose for HOMA-IR and QUICKI at 140 mg/dL in this study ( 13 ). As the number of subjects with Mets in 2011 was 510, the final target subject became 2642 (Figure 1). 

Informed consent was obtained from the study participants, and the study protocol was approved by the ethics committee of the university.

Data on lifestyle-related variables was gathered from the self-administered questionnaire. Smoking habit was categorized as current smoking (0) or no smoking, including ex-smoking (1). Drinking habit was categorized as everyday drinking (0) or occasional drinking, including no drinking (1). Physical activity was categorized as everyday exercise, including walking for ≥ 1 hour (1) or no daily exercise habit (0). Especially, I used the following questionnaire to evaluate habitual exercise; “Do you take exercise every day, including walking for at least one hour?”

The author used the criteria of the National Cholesterol Education Program Adult Treatment Panel III (ATP III), in which MetS is defined based on the presence of three or more of the following criteria: central obesity (waist circumference ≥ 85 cm) (14); hypertriglyceridemia (serum triglyceride ≥ 150 mg/dL); reduced serum level of high-density lipoprotein (HDL) cholesterol (serum HDL < 40 mg/dL); high-blood pressure (systolic BP ≥ 130 mmHg and/or a diastolic BP ≥ 85 mm Hg); high fasting plasma glucose (FPG) (≥ 100 mg/dL) (15).

Serum ALT, GGT, and uric acid were measured with an automatic analyzer (7700 series, Hitachi, Tokyo). Serum high-sensitivity CRP measurement was based on a Latex turbidity assay (Mitsubishi Kagaku Iatron, Tokyo, Japan) using the Hitachi 7700 auto-analyzer. The detection limit of this assay is 0.1 mg/L. The intraassay CVs for repeated measurements ranged from 0.84% to 2.54%. Insulin was measured using CLEIA (Fujirebio Inc, Tokyo, Japan) and Lumipulse Presto II. The detection limit of this assay is 0.3 mIU/L. The intraassay CV for repeated measurements was 3.06%. Serum HDL cholesterol, triglyceride, and glucose levels were determined enzymatically with a Hitachi 7700 auto-analyzer.

As the insulin-related biomarkers, HOMA-IR and QUICKI were calculated as follows:

HOMA-R = (FPG × insulin)/405; QUICKI = 1/ (common logarithms (FPG × insulin))

The units of glucose and insulin for the HOMA-R calculation were mg/dL and mIU/L, respectively.

Mathematically, the association between these two indicators becomes a monotone decreasing function. The distribution of HOMA-IR is logarithmic-normal; whereas, that of QUICKI is normal. Although FPG was used for the calculation, the serum insulin and HOMA-IR were closely related (16).

All the statistical analyses were conducted using SPSS 16.0 for Windows (SPSS Japan, Tokyo). Spearman's rho was calculated for the univariate analysis. Then, a multiple logistic regression analysis was performed for predicting the risk of MetS development as the dependent variable. Three lifestyle factors were originally classified in a binary manner. Other independent variables were also converted to binary format, namely, age (≥ 45 and <45 years old), uric acid (≥ 7 and <7 mg/dL), ALT (≥ 45 and < 45 IU/L), GGT (≥ 50 and < 50 IU/L), CRP (≥ 1 and <1 mg/L), HOMA-IR (≥ 2.5 and < 2.5), and QUICKI (≤ 0.33 and >0.33), which were assigned the values of 1 and 0. A cut-off value of the highest tertile of age became 45 years, and cut-off value of CRP was adopted as "mildly elevated" (17, 18). P-values of less than 0.05 according to a two-tailed test were considered to denote statistical significance.

## 4. Results

Characteristics of the study population at baseline were presented in Table 1, and the correlation matrix was also presented in Table 2. The analysis revealed that HOMA-IR and QUICKI were mathematically equal. In addition, strong associations were observed between the serum insulin and HOMA-IR or QUICKI, with Spearman’s rho of 0.98 and -0.98, respectively. 

**Table 1.** Characteristics of the Study Population (n=2642)

Variables	Results
**Age, y**	43.2 ± 6.5 ^b^
**Waist circumference, cm**	81.7 ± 8.6 ^b^
**Systolic blood pressure, mmHg**	123.7 ± 12.0 ^b^
**Diastolic blood pressure, mmHg**	77.4 ± 9.4 ^b^
**Triglyceride, mg/dL**	110.8 ± 72.5 ^b^
**HDL cholesterol, mg/dL**	61.2 ± 15.3 ^b^
**Plasma glucose, mg/dL**	91.5 ± 9.1 ^b^
**Uric acid, mg/dL**	6.0 ± 1.2 ^b^
**HOMA-IR**^a^	1.3 (1.8)
**CRP, mg/L**^a^	0.4 (2.7)
**ALT, IU/L**^a^	24.5 (1.7)
**GGT, IU/L**^a^	30.9 (1.9)
**No[Table-fn fn5306]smoking or ex-smoking**	52.7% (1392/2642)
**Exercise for ≥ 1 hour everyday**	37.4% (989/2642)
**Not everyday drinking**	66.0% (1743/2642)

^a^Geometric mean (geometric standard deviation)

^b^Mean ± SD

Abbreviations: HDL; high-density lipoprotein, ALT; alanine aminotransferase, GGT; gamma-glutamyl transferase, CRP; C-reactive protein, HOMA-IR; the homeostasis model assessment for insulin

**Table 2.** Correlation Matrix of Variables Relating to Glucose Metabolism at Baseline Study (N = 2642)

Spearman’s rho^a^	FPG	Insulin	HOMA-IR[Table-fn fn5308]
**FPG**			
**Insulin**	0.16		
**HOMA-IR**	0.32	0.98	
**QUICKI**	-0.32	-0.98	-1.0

^a^There were significant associations among four variables with significance level of 0.01.

Abbreviations: FPG; fasting plasma glucose, HOMA-R; the homeostasis model assessment for insulin resistance, QUICKIl; the quantitative insulin sensitivity check index

The incidence of metabolic syndrome was 8.8% after one-year follow-up. Unadjusted and age-adjusted odds ratios (95% confidence intervals) of HOMA-IR (≥ 2.5) for MetS were 2.9 (2.1 - 4.0) and 3.0 (2.2 - 4.1), respectively (P < 0.001). In addition, unadjusted and age-adjusted odds ratios (95% confidence intervals) of QUICKI (≤ 0.33) for MetS were 3.2 (2.3 - 4.5) and 3.3 (2.3 - 4.6), respectively (P < 0.001). A multiple logistic regression analysis showed that absence of regular physical activity, age (≥ 45 years old), serum uric acid (≥ 7 mg/dL), serum alanine aminotransferase (≥ 45 IU/L), serum C-reactive protein (≥ 0.1 mg/L), and HOMA-IR (≥ 2.5) Were identified as significantly contributing to the risk of MetS, with odds ratios (95% confidence intervals) of 0.68 (0.50 – 0.92), 2.0 (1.5 – 2.6), 2.2 (1.6 – 3.0), 1.5 (1.02 – 2.2), 1.4 (1.01 – 2.0), and 2.3 (1.6 – 3.3) respectively (Table 3). 

**Table 3.** Odds Ratios and 95% Confidence Intervals of Several Factors to Predict Metabolic Syndrome by Logistic Regression Analysis

Variables	Positive [Table-fn fn5309]Category	OR (95%CI)	Significance
**Age**	45 years old or higher	2.0 (1.5, 2.6)	P < 0.001
**Uric acid**	7 mg/dL or higher	2.2 (1.6, 3.0)	P < 0.001
**ALT**	45 IU/L or higher	1.5 (1.02, 2.2)	P < 0.05
**GGT**	50 IU/L or higher	1.4 (0.99, 2.0)	ns
**CRP**	1 mg/L or higher	1.4 (1.01, 2.0)	P < 0.05
**HOMA-IR**	≥2.5	2.3 (1.6, 3.3)	P < 0.001
**Smoking**	No smoking or ex-smoking	1.02 (0.77, 1.3)	ns
**Exercise**	≥ 1 hour everyday	0.68 (0.50, 0.92)	P < 0.05
**Drinking**	Not everyday drinking	1.01 (0.74, 1.4)	ns

Abbreviations: ALT; alanine aminotransferase, GGT; gamma-glutamyl transferase, CRP; C-reactive protein, HOMA-IR; the homeostasis model assessment for insulin resistance

When QUICKI was used instead of HOMA-IR, the absence of regular physical activity, age (≥ 45 years old), serum uric acid (≥ 7 mg/dL), serum gamma-glutamyl transferase (≥ 50 IU/L), and QUICKI (≤ 0.33) were identified as significantly contributing to the risk of MetS, with odds ratios (95% confidence intervals) of 0.68 (0.51 – 0.93), 2.0 (1.5 – 2.6), 2.2 (1.6 – 3.0), 1.4 (1.01 – 2.0), and 2.5 (1.7 – 3.6) respectively (Table 4). 

**Table 4.** Odds Ratios and 95% Confidence Intervals of Several Factors to Predict Metabolic Syndrome by Logistic Regression Analysis

Variables	Positive[Table-fn fn5310]Category	OR (95%CI)	Significance
**Age**	45 years old or higher	2.0 (1.5, 2.6)	P < 0.001
**Uric acid**	7 mg/dL or higher	2.2 (1.6, 3.0)	P < 0.001
**ALT**	45 IU/L or higher	1.5 (0.99, 2.1)	ns
**GGT**	50 IU/L or higher	1.4 (1.01, 2.0)	P <0.05
**CRP**	1 mg/L or higher	1.4 (0.997, 1.9)	ns
**QUICKI**	≤ 0.33	2.5 (1.7, 3.6)	P < 0.001
**Smoking**	No smoking or ex-smoking	1.02 (0.77, 1.4)	ns
**Exercise**	≥ 1 hour everyday	0.68 (0.51, 0.93)	P < 0.05
**Drinking**	Not everyday drinking	1.02 (0.75, 1.4)	ns

ALT; alanine aminotransferase, GGT; gamma-glutamyl transferase, CRP; C-reactive protein, QUICKI; the quantitative insulin sensitivity check index

## 5. Discussion

The predictive ability of insulin resistance or insulin sensitivity for MetS in Japanese male subjects was checked by a one-year follow-up study. HOMA-IR and QUICKI were equally contributed to the incidence of MetS after adjustments for other independent variables.

WHO defined insulin resistance as a HOMA-IR of ≥ 1.8 (19). In this study, the author adopted another criterion for insulin resistance in patients with HOMA-IR ≥ 2.5, which is popular in Japan. The author also adopted QUICKI ≤ 0.33 as a criterion of insulin sensitivity. The results of the study showed a strong association between the serum insulin and HOMA-IR or QUICKI in this study. This means that insulin-related biomarkers can be used interchangeably; although, there is a limitation that both HOMA-IR and QUICKI can only be applied reliably for subjects with fasting glucose levels under 140 mg/dL. The author previously reported that there was a high correlation of insulin with HOMA and QICKI, but low correlation with FPG (16). In this study, the same finding was observed and contribution of FPG to HOMA-IR and QUICKI was relatively small.

Among the lifestyle factors, only habitual exercise contributed significantly to lowering the risk of MetS in this study, which was consistent with a previous report (20). Smoking is known to be associated with insulin resistance and the risk of MetS development (21-23); however, our present results did not corroborate this notion. This discrepancy should be explored by further follow-up of the target population. By logistic regression analysis, physical activity was identified as a low-risk factor, as it was associated with a reduction in the incidence of MetS by 32%.

Serum ALT has been reported to be associated with adiposity and insulin resistance (24), and serum GGT to be a predictor of cardiovascular mortality (25). In my study, serum ALT or GGT was selected as a significant contributor to the risk of MetS when HOMA-IR or QUICKI was used as an independent variable, respectively. However, as the P value was near 0.05, the contribution was not statistically strong for either case in this study. The findings were similar for serum CRP, which has been found to contribute significantly to the risk of MetS. In contrast, serum uric acid was found to be significantly associated with the risk of MetS, its significance being equivalent to that of age. This finding was consistent with the results of a long-term follow-up study suggesting that serum uric acid was associated with the risk of MetS and cardiovascular mortality (26).

Categorization of continuous variable leads to the loss of power. But understanding the level of risk improves by categorization. As simplest categorization is the binary classification, the author adopted binary conversion of independent variable for logistic regression analysis.

This study was a one-year follow-up study, and the causality could only be partially determined. But the two insulin-related biomarkers examined exhibited equivalent ability as predictive markers for MetS in this study.

## References

[1] Norris JM, Rich SS (2012). Genetics of glucose homeostasis: implications for insulin resistance and metabolic syndrome.. Arterioscler Thromb Vasc Biol..

[2] Romeo GR, Lee J, Shoelson SE (2012). Metabolic syndrome, insulin resistance, and roles of inflammation--mechanisms and therapeutic targets.. Arterioscler Thromb Vasc Biol..

[3] Kawamoto R, Tabara Y, Kohara K, Miki T, Kusunoki T, Takayama S (2010). High-sensitivity C-reactive protein and gamma-glutamyl transferase levels are synergistically associated with metabolic syndrome in community-dwelling persons.. Cardiovasc Diabetol..

[4] Mason JE, Starke RD, Van Kirk JE (2010). Gamma-glutamyl transferase: a novel cardiovascular risk biomarker.. Prev Cardiol..

[5] Jacobs M, van Greevenbroek MM, van der Kallen CJ, Ferreira I, Feskens EJ, Jansen EH (2011). The association between the metabolic syndrome and alanine amino transferase is mediated by insulin resistance via related metabolic intermediates (the Cohort on Diabetes and Atherosclerosis Maastricht [CODAM] study).. Metabolism..

[6] Tomiyama H, Higashi Y, Takase B, Node K, Sata M, Inoue T (2011). Relationships among hyperuricemia, metabolic syndrome, and endothelial function.. Am J Hypertens..

[7] Yamasaki T, Tomita K (2008). [Relationship between hyperuricemia and metabolic syndrome].. Nihon Rinsho..

[8] Yang T, Chu CH, Bai CH, You SL, Chou YC, Chou WY (2012). Uric acid level as a risk marker for metabolic syndrome: a Chinese cohort study.. Atherosclerosis..

[9] Matthews DR, Hosker JP, Rudenski AS, Naylor BA, Treacher DF, Turner RC (1985). Homeostasis model assessment: insulin resistance and beta-cell function from fasting plasma glucose and insulin concentrations in man.. Diabetologia..

[10] Muniyappa R, Lee S, Chen H, Quon MJ (2008). Current approaches for assessing insulin sensitivity and resistance in vivo: advantages, limitations, and appropriate usage.. Am J Physiol Endocrinol Metab..

[11] Wallace TM, Matthews DR (2002). The assessment of insulin resistance in man.. Diabetic Medicine..

[12] Yokoyama H, Emoto M, Fujiwara S, Motoyama K, Morioka T, Komatsu M (2004). Quantitative insulin sensitivity check index and the reciprocal index of homeostasis model assessment are useful indexes of insulin resistance in type 2 diabetic patients with wide range of fasting plasma glucose.. J Clin Endocrinol Metab..

[13] DeFronzo Ralph A, Ferrannini Eleuterio, Simonson Donald C (1989). Fasting hyperglycemia in non-insulin-dependent diabetes mellitus: Contributions of excessive hepatic glucose production and impaired tissue glucose uptake.. Metabolism..

[14] Matsuzawa Y, Funahashi T, Nakamura T (2011). The concept of metabolic syndrome: contribution of visceral fat accumulation and its molecular mechanism.. J Atheroscler Thromb..

[15] Alberti KGeorge MM, Zimmet Paul, Shaw Jonathan (2005). The metabolic syndrome—a new worldwide definition.. The Lancet..

[16] Kawada T (2010). Preliminary report: homeostasis model assessment of insulin resistance, an indicator of insulin resistance, is strongly related to serum insulin: practical data presentation and the mathematical basis.. Metabolism..

[17] Arima H, Kubo M, Yonemoto K, Doi Y, Ninomiya T, Tanizaki Y (2008). High-sensitivity C-reactive protein and coronary heart disease in a general population of Japanese: the Hisayama study.. Arterioscler Thromb Vasc Biol..

[18] Pearson TA, Mensah GA, Alexander RW, Anderson JL, Cannon RO, 3rd, Criqui M (2003). Markers of inflammation and cardiovascular disease: application to clinical and public health practice: A statement for healthcare professionals from the Centers for Disease Control and Prevention and the American Heart Association.. Circulation..

[19] Alberti KGMM, Zimmet PZ (1998). Definition, diagnosis and classification of diabetes mellitus and its complications. Part 1: diagnosis and classification of diabetes mellitus. Provisional report of a WHO Consultation.. Diabetic Medicine..

[20] Li Y, Yatsuya H, Iso H, Tamakoshi K, Toyoshima H (2010). Incidence of metabolic syndrome according to combinations of lifestyle factors among middle-aged Japanese male workers.. Prev Med..

[21] Oh SW, Yoon YS, Lee ES, Kim WK, Park C, Lee S (2005). Association Between Cigarette Smoking and Metabolic Syndrome: The Korea National Health and Nutrition Examination Survey.. Diabetes Care..

[22] Onat A, Can G, Cicek G, Dogan Y, Kaya H, Gumrukcuoglu HA (2012). Diverging sex-specific long-term effects of cigarette smoking on fasting insulin and glucose levels in non-diabetic people.. Clin Biochem..

[23] Tonstad S, Svendsen M (2005). Premature coronary heart disease, cigarette smoking, and the metabolic syndrome.. Am J Cardiol..

[24] Patel DA, Srinivasan SR, Xu JH, Chen W, Berenson GS (2007). Persistent elevation of liver function enzymes within the reference range is associated with increased cardiovascular risk in young adults: the Bogalusa Heart Study.. Metabolism..

[25] Ruttmann E, Brant LJ, Concin H, Diem G, Rapp K, Ulmer H (2005). Gamma-glutamyltransferase as a risk factor for cardiovascular disease mortality: an epidemiological investigation in a cohort of 163,944 Austrian adults.. Circulation..

[26] Fang J, Alderman MH (2000). Serum uric acid and cardiovascular mortality the NHANES I epidemiologic follow-up study, 1971-1992. National Health and Nutrition Examination Survey.. JAMA..

